# Ticagrelor reduces doxorubicin-induced pyroptosis of rat cardiomyocytes by targeting GSK-3β/caspase-1

**DOI:** 10.3389/fcvm.2022.1090601

**Published:** 2023-01-06

**Authors:** Shu-hui Wang, Meng-jin Sun, Si-yue Ding, Chun-li Liu, Jing-min Wang, Sheng-na Han, Xi Lin, Qian Li

**Affiliations:** ^1^Department of Ultrasound, The Affiliated Cancer Hospital of Zhengzhou University, Henan Cancer Hospital, Zhengzhou, China; ^2^Department of Pharmacology, School of Basic Medical Sciences, Zhengzhou University, Zhengzhou, China; ^3^State Key Laboratory of Oncology in South China, Department of Ultrasound, Sun Yat-sen University Cancer Center, Guangzhou, China

**Keywords:** doxorubicin, pyroptosis, ticagrelor, GSK-3β, caspase-1

## Abstract

Doxorubicin (Dox) is a widely used clinical drug whose cardiotoxicity cannot be ignored. Pyroptosis (inflammatory cell death) has gradually gained attention in the context of Dox-induced cardiotoxicity. In addition to the inhibition of platelet activation by ticagrelor, little is known about its other pharmacological effects. Glycogen synthase kinase 3β (GSK-3β) has been shown to contribute to the pathological process of pyroptosis, but whether it is related to the potential role of ticagrelor is unclear. In this study, we investigated the effects of ticagrelor on Dox-induced pyroptosis in cardiomyocytes. Rats were treated with ticagrelor (7.5 mg/kg, i.g.) 1 h before intravenous injection of Dox (2.5 mg/kg), once every 3 days, six times in total. Hearts were collected for histochemical analysis and western blot detection 8 weeks after the last administration. Ticagrelor was shown to significantly improve cardiac function by inhibiting GSK-3β/caspase-1/GSDMD activation. *In vitro* experiments were conducted using rat cardiac myocytes (RCMs) and rat embryonic cardiac-derived H9c2 cells. Pretreatment with ticagrelor (10 μm) significantly inhibited Dox (1 μm)-induced hypertrophy and reversed the upregulation of GSDMD-NT expression. We showed that ticagrelor suppressed the activation of Akt caused by Dox in the heart tissue as well as in RCMs/H9c2 cells caused by Dox. When GSK-3β expression was absent in H9c2 cells, the inhibitory effect of ticagrelor on Dox-induced caspase-1/GSDMD activation was weakened. These data showed that ticagrelor reduced Dox-induced pyroptosis in rat cardiomyocytes by targeting GSK-3β/caspase-1.

## Introduction

Doxorubicin (Dox), a chemotherapeutic drug used to treat malignant tumors, is a non-selective class I anthracycline drug (1). Dox, an inhibitor of DNA topoisomerase II, causes DNA damage in cancer cells and is widely used in cancer chemotherapy (2). Because it is indispensable in clinical treatment, the cumulative dose of Dox impairs cardiac function in up to a quarter of patients, eventually leading to heart failure (HF) (3). Similar to the side effects of other drugs, the cardiotoxic reaction of Dox was initially considered acute. However, some studies demonstrate that Dox-induced cardiotoxicity is persistent, resulting in myocardial cell damage and the progressive decline of cardiac function, gradually leading to obvious HF (4, 5). Excessive reactive oxygen species (ROS) production, topoisomerase 2β (TOP2β) inhibition, and mitochondrial damage are the three main factors leading to cardiomyocyte cell death (6). Although the mechanism of Dox-induced cardiotoxicity has been widely studied, the molecular pathogenesis is not completely understood.

HF therapy in patients with Dox-induced cardiotoxicity has not yet been thoroughly investigated. Dexrazoxane, which binds to TOP2β, is the only FDA-approved drug for sensitive cardiac patients (7). Angiotensin inhibitors and β-blockers show protective action over Dox-induced cardiotoxicity (4, 8). More powerful agents must be explored to address this troublesome problem. Ticagrelor is an oral, first-line antiplatelet drug (9). In addition to its antiplatelet activity, increasing evidence suggests that ticagrelor has other pharmacological functions. Ticagrelor has been reported to help reduce the mortality of inflammation-related diseases such as sepsis and infection compared with clopidogrel (10, 11). Another report showed that ticagrelor could reduce interleukin-6 (IL-6) and tumor necrosis factor-α (TNF-α) levels in diabetic patients with acute coronary syndrome (ACS) (12). In diabetic rats with ischemia-reperfusion injury, ticagrelor combined with rosuvastatin reduced the mRNA levels of IL-1β, IL-6, and NLRP3 in the rat myocardium (13). Therefore, the positive effects of ticagrelor on Dox-induced cardiotoxicity are worth exploring.

Since pyroptosis was first confirmed in 2001, its role in the pathogenesis of cardiovascular diseases has been widely confirmed (14). Pyroptosis is an inflammatory programmed cell death that is usually accompanied by the activation of caspase-1/3/4/11, which cleaves gasdermin D (GSDMD) to form the GSDMD-NT. GSDMD-NT migrates and interacts with the cell membrane by lipid binding site, followed by GSDMD-NT oligomerization, resulting in the pore forming, cell pyroptosis, and cytokine release. Moreover, the pores also lead to severe leakage of liposomes as well as dissolution of membranes including cellular membranes and organelle membranes (15, 16). NLRP3 inflammasome formation in cardiomyocytes has potential to activate caspase 1 and induces pyroptosis. NLRP3 inflammasome activation generally includes two processes: a priming event that induces transcription of NLRP3 and precursors of pro-caspase-1 and pro-IL-1β via toll-like receptor (TLR)/NF-κB signaling, and a subsequent assembly of NLRP3 with the adaptor protein apoptosis-associated speck-like protein containing a caspase recruitment domain (ASC) and pro-caspase-1 (17). The regulatory mechanism upstream of pyroptosis is key to preventing or reversing its occurrence.

Base on the observation of GSK-3β null mice embryogenesis, it implies that GSK-3β modulation participates important role of heart physical functions and related diseases (18). Our recent study revealed that GSK-3β regulate pyroptosis (19). GSK-3β contributes to Dox-induced cardiotoxicity by mediating oxidative stress, inflammation and apoptosis (20). Therefore, we want to determine whether GSK-3β mediated pyroptosis is the key to DOX induced cardiotoxicity. Furthermore, we want to confirm whether ticagrelor reduces DOX induced cardiotoxicity through GSK-3β.

## Materials and methods

### Animal protocol

Male Wister rats weighing 220 ± 10 g were obtained from Vital River Laboratory Animal Technology Co., Ltd. (Beijing, China). All rats were kept in the Animal Experiments Committee of Zhengzhou University (Ethics No. ZZULAC20211112[06]) at 24°C with a 12-h light/dark cycle and free access to food and water. This study conformed to the Guide for the Care and Use of Laboratory Animals (NIH Publication No. 85-23, revised 1996).

To induce cardiotoxicity, the rats were injected with a cumulative dose of 15 mg/kg Dox (HY-15142, Med Chem Express, Monmouth Junction, NJ, USA) or vehicle via three weekly injections (2.5 mg/kg i.p. once every 3 days). To investigate the role of ticagrelor *in vivo*, 1 h before each Dox injection, the rats were injected with 7.5 mg/kg ticagrelor (i.g., HY-10064, Med Chem Express). There were 12 rats in each group, 36 rats in total. Subsequent analyses were performed 8 weeks after the last injection. At the end of the experiment, six rats per group were sacrificed to make heart slices and other rats heart and blood samples were collected.

### Rat echocardiography

Rat echocardiography was performed under isoflurane anesthesia using a Visualsonics imaging system under M-mode (Vivo 2100, Toronto, Canada). General anesthesia was initially induced with 5% isoflurane oxygen and then reduced to 1.5% to maintain anesthesia. Left ventricular fractional shortening (LVFS), left ventricular ejection fraction (LVEF), left ventricular end-systolic diameter (LVESD), and left ventricular end-diastolic diameter (LVEDD) were measured using software included in the Visualsonics system.

### Histologic analysis

Rats heart were washed with cold saline and placed in 4% paraformaldehyde at 25°C for 24 h. Four micrometers sections were collected and subjected to hematoxylin and eosin (HE), Masson’s trichrome, and wheat germ agglutinin (WGA) (#L4895; Sigma Aldrich, USA) staining. An optical microscope (BX60; Olympus Corporation, Tokyo, Japan) was used to observe stained sections. Images were analyzed using ImageJ Launcher 1.8.0 (National Institutes of Health, Bethesda, MD, USA).

### Immunofluorescent staining

Heart sections (4 μm) were fixed with 4% paraformaldehyde, blocked with 10% fetal bovine serum (FBS, #35-081-CV, Corning Inc., Corning, NY, USA), and permeabilized with Triton X-100. Anti-p-Ser9-GSK-3β (1:200, #93235, Cell Signaling Technology, Danvers, MA, USA), anti-cleaved-capsase-1 (1:200, #PA5-99390, Invitrogen, Carlsbad, CA, USA), anti-GSDMD-NT (1:200, #PA5-115330, Invitrogen) and anti-ASC (1:200, #6741R-A5555, Bioss) antibodies were incubated overnight at 4°C, followed by secondary antibodies conjugated to Alexa Fluor 680 (1:500, #A10043, Invitrogen) and 4’,6-diamidino-2-phenylindole dihydrochloride (DAPI) (#S2110, Solarbio, China). Anti-α-actinin was purchased from Abcam (Cambridge, MA, USA, 1:200, #ab68194). All slices were analyzed using the EVOS M7000 Imaging System (Thermo Fisher Scientific, Waltham, MA, USA).

### Immunohistochemical staining

After heart sections (4 μm) were fixed, blocked and permeabilized, anti-IL-1β (1:200, #PA5-105048, Invitrogen) and anti-IL-18 (1:200, #ab191860, Abcam) antibodies were used to incubate the slices for 2 h, followed by incubation with secondary antibodies conjugated to AlexaFluor 488 (1:200; Invitrogen) or AlexaFluor 594 (1:200; Invitrogen) at 37°C for 1 h. All slices were analyzed using the EVOS M7000 Imaging System (Thermo Fisher Scientific, Waltham, MA, USA).

### Serum lactate dehydrogenase (LDH), creatine kinase-MB (CK-MB), troponin (cTnI), brain natriuretic peptide (BNP), IL-1β, and IL-18 levels

Serum levels of LDH, CK-MB, cTnI, and BNP levels were assessed using standardized commercially available kits (Jian Cheng, Nanjing, China). Serum IL-1β and IL-18 levels were assessed using rat enzyme-linked immunoassay (ELISA) kits (Abcam) according to the manufacturer’s instructions. The information of the above kits were shown in Table 1.

**TABLE 1:** List of technical parameters of the kits.

	Cat. no.	Linear range	Intra CV (%)	Inter CV (%)
LDH	A020-2-2	9.0–5,000 U/L	2.7	4.9
CK-MB	197-1-2	10.0–2,000 U/L	3.6	5.3
cTnI	E019-1-1	15.0–1,000 pg/ml	4.4	5.7
BNP	H166	10.0–1,000 pg/ml	5.1	7.7
IL-1β	255730	54.69–3,500 pg/ml	3.9	4.5
IL-18	213909	15.6–1,000 pg/ml	5.5	7.3

### Cell culture

Both primary rat cardiomyocytes (RCMs) and rat embryonic cardiac-derived H9c2 cells were cultured in Dulbecco’s modified Eagle’s medium (DMEM) supplemented with 10% FBS and 1% penicillin/streptomycin (#P1400, Solarbio, Beijing, China).

Clean heart ventricles were digested with 0.1 mg/ml trypsin (#T1300, Solarbio, Beijing, China) and 0.1 mg/ml collagenase II (#LS004176, Worthington, Lakewood, NJ, USA) from 1-day-old rat hearts for RCMs. RCMs were seeded in 6-well plates at 70–90% confluency and incubated for 24 h in medium containing various combinations of the following reagents: 1/10/20 μm ticagrelor and 0.1/1/10 μm Dox. All inhibitors were dissolved in 0.1% DMSO (#D8370, Solarbio) and an equivalent quantity of DMSO was used as a control.

H9c2 cells were purchased from ATCC (Manassas, VA, USA). When the cells reached 70–90% confluence, they were incubated for 24 h in medium containing the following reagents: 10 μm MCC950 (inhibitor of NLRP3, #HY-12815, MedChemExpress), 10 μm MK2206 (inhibitor of Akt, #HY-10358, MedChemExpress), 20 μm TDZD-8 (inhibitor of GSK-3β, #HY-11012, MedChemExpress), 25 μm VX765 (inhibitor of caspase-1, #HY-13205, MedChemExpress), 10 μm ticagrelor, and 1 μm Dox. All the inhibitors were dissolved in 0.1% DMSO, and an equivalent quantity of DMSO was used as a control.

### Cell viability assay

RCMs were seeded in a 96-well plate. After culturing for 24 h with different pharmaceutical ingredients, 10 μl MTT (0.5 mg/ml, #HY-15924, MedChemExpress) was added and the cells were incubated at 37°C for 4 h. The culture medium was removed and DMSO was added. The absorbance was measured at 570 nm using a microplate reader (Thermo Fisher Scientific).

### Propidium iodide (PI) staining and lactate dehydrogenase (LDH) release

RCMs and H9c2 cells were seeded in 24-well plates and PI dissolved in 0.1% DMSO (2 μg/ml, MedChemExpress) was added. Images of the pyroptotic cells were captured using a Nikon Ti2 microscope (Nikon, Minato Ward, Tokyo, Japan) after 2 h.

The release of LDH into the supernatants was measured using the CyQUANT LDH Cytotoxicity Assay Kit (#C20301, Invitrogen) according to the manufacturer’s instructions.

### Phalloidin staining

H9c2 cells were seeded in 35 mm confocal dishes. After incubation with the ingredients, H9c2 cells were fixed with 4% paraformaldehyde for 2 h. Next, 2 mg/ml glycine was added to neutralize residual paraformaldehyde. The culture medium was discarded and the cells were washed with PBS. 0.5 μm rhodamine-labeled phalloidin incubated the dishes for 30 min. The cells were incubated with DAPI (0.5 M DAPI incubated them for 10 min. The cells were examined under an Eclipse E100 fluorescence microscope (Nikon, Japan).

### Cell transfection

Rat GSK-3β shRNA and negative control shRNA were constructed by Hanbio (Wuhan, China). H9c2 cells were seeded in 6-well plates and transfected with 2.5 μg of shRNAs using 5 μl Lipofectamine 3000 (Invitrogen). The sequences of the specific shRNAs are listed in Table 2.

**TABLE 2:** List of shRNAs sequences.

	Forward (5′-3′)	Reverse (5′-3′)
shNC	GATCCGTTCTCCGAACGTGTCACGTAATTCAAGAG ATTACGTGACACGTTCGGAGAATTTTTTC	AATTGAAAAAATTCTCCGAACGTGTCACGTAAT CTCTTGAATTACGTGACACGTTCGGAGAACG
shGSK-3β/1	GATCCGGTAGCATGAAAGTTAGCAGAGATATTCAAGA GATATCTCTGCTAACTTTCATGCTACCTTTTTTG	AATTCAAAAAAGGTAGCATGAAAGTTAGCAGAGA TATCTCTTGAATATCTCTGCTAACTTTCATGCTACCG
shGSK-3β/2	GATCCGCAAAGGCCATCTGCATATCTCTCGAG AGATATGCAGATGGCCTTTGCTTTTTG	AATTCAAAAAGCAAAGGCCATCTGCA TATCTCTCGAGAGATATGCAGATGGCCTTTGC

### Adeno-associated virus (AAV) 9 viral delivery protocol

The AAV9-shGSK-3β and AAV9-shNC constructs were constructed by Hanbio, using shRNAs (Table 2). The viral vector was injected into the heart after opening the thoracic cavity of rats. Two weeks before drug administration, AAV9 vectors (50 μl) at a dose of 1 × 10^11^ viral genome particles were randomly injected into the left ventricle of the rats. These virus solutions were distributed to five sites. A single injection was administered to the left ventricular apex. Double injections at a spacing of 5 mm were performed in the anterior and lateral walls of the left ventricle. There were 12 rats in each group, 36 rats in total. At the end of the experiment, six rats per group were sacrificed to make heart slices and other rats heart and blood samples were collected.

### Western blot analysis

Proteins from rat heart tissues and cell lysates were collected. Western blotting was performed using the standard methods. Membranes were incubated with specific antibodies against GSK-3β (1:1,000, #12456, Cell Signaling Technology), p-Ser9-GSK-3β (1:1,000), cleaved-capsase-1 (1:1,000), GSDMD-NT (1:1,000), NLRP3 (1:1,000, #ab263899, Abcam), ASC (1:1,000), p-Ser473-Akt (1:1,000, #4060, Cell Signaling Technology), Akt (1:1,000, #9272, Cell Signaling Technology), PARP (1:1,000, #191217, Abcam), caspase-3 (1:1,000, #184787, Abcam) and GAPDH (1:10,000, # 60004-1, Proteintech). The band intensities were analyzed using ImageJ.

### Statistical analysis

Data are presented as the mean ± SD and were analyzed using SPSS (version 21.0; SPSS Inc., Chicago, IL, USA). Kolmogorov-Smirnov test was used to check whether the samples conform to the normal distribution. One-way analysis of variance followed by the Bonferroni *post-hoc* test was used to compare multiple groups. *P*-values less than 0.05 were considered statistically significant.

## Results

### Ticagrelor attenuates Dox-induced rat cardiac dysfunction

**FIGURE 1:**
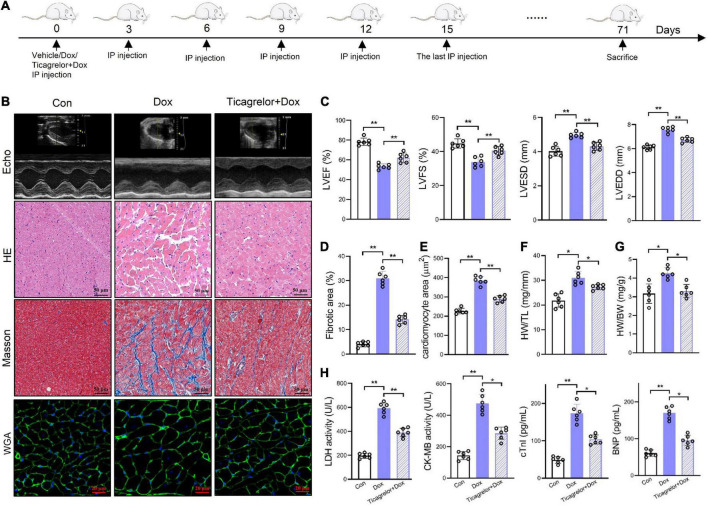
Ticagrelor attenuates Dox-induced rat cardiac dysfunction. **(A)** Schematic illustration of Dox induced cardiac toxicity in rats treated with ticagrelor pre-administration. **(B)** Representative echocardiographic (Echo), HE, Masson’s, and WGA staining images. **(C)** Cardiac function index for LVEF, LVFS, LVESD, and LVEDD (*n* = 6). **(D)** Fibrosis area of rat heart sections (*n* = 6). **(E)** Quantification of the cardiomyocyte hypertrophy (*n* = 6). **(F,G)** The ratios of HW/BW **(F)** and HW/TL **(G)** in rats (*n* = 6). **(H)** LDH, CK-MB, cTnI, and BNP levels in rats serum (*n* = 6). *p < 0.05, **p < 0.01.

We used Dox to induce cardiotoxicity in rats and observed the effects of ticagrelor (Figure 1A). Echocardiographic analysis (Figure 1B) showed that Dox administration decreased LVEF and LVFS in rats, along with increased LVESD and LVEDD (Figure 1C) at the end of the experiments. Ticagrelor treatment significantly attenuated the LV dysfunction. Dox-induced heart lesions were confirmed by HE and Masson’s trichrome staining (Figure 1B). The morphology and arrangement of cardiac cells in the Con group were normal, while the cardiac structure in Dox group was damaged with obvious fiber breakage. Ticagrelor pretreatment reduced the area of fibrosis induced by Dox from 30.9 to 14.1% (Figures 1B, D). In addition, WGA staining (Figures 1B, E), the heart weight/body weight ratio (HW/BW) (Supplementary Table 1 and Figure 1F) and heart weight per tibia length (HW/TL) (Figure 1G) in rats confirmed that Dox induced cardiac hypertrophy, which could be improved by ticagrelor. As for cardiac-specific parameters, Dox significantly increased the serum level of LDH, CK-MB, cTnI, and BNP and ticagrelor suppressed their abnormality (Figure 1H).

### Ticagrelor inhibits GSK-3β-mediated pyroptosis in rats treated by Dox

**FIGURE 2:**
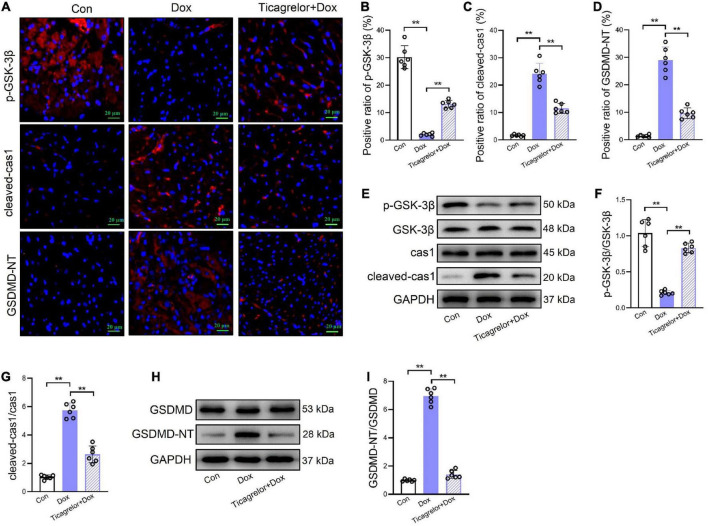
Ticagrelor inhibits GSK-3β-mediated pyroptosis in rats treated by Dox. **(A–D)** Representative images and positive ratio of p-GSK-3β **(A,B)**, cleaved-cas1 **(A,C)**, and GSDMD-NT **(A,D)** under immunofluorescence (*n* = 6). Cleaved-cas1: cleaved-caspase-1. **(E–G)** Representative immunoblots and the corresponding quantification of the ratio of p-GSK-3β/GSK-3β and cleaved-cas1/cas1 in heart tissues (*n* = 6). **(H,I)** Representative immunoblots and the corresponding quantification of GSDMD-NT/GSDMD in heart tissues (*n* = 6). **p < 0.01.

Ticagrelor effectively prevented Dox-induced cardiac function damage in rats. Is this effect related to pyroptosis? How can pyroptosis be regulated? We observed pyroptosis in rat heart tissues. We performed immunofluorescence staining on rat heart sections and found that ticagrelor reduced the number of GSDMD-NT-positive cardiomyocytes induced by Dox (Figures 2A, D). Moreover, the expression of GSDMD-NT in the myocardial tissue protein extract showed a consistent trend (Figures 2H, I).

Pyroptosis mainly occurs via the canonical caspase-1-dependent pathway, and we examined whether Dox caused pyroptosis in rat cardiomyocytes through the activation of caspase-1. GSK-3β, has been proved to play a role in Dox induced cardiotoxicity (20, 21). Our results showed that after Dox administration, the proportion of p-GSK-3β positive heart slices decreased (Figures 2A, B) and cleaved-caspase-1 increased (Figures 2A, C). Western blot analysis showed that Dox decreased the p-GSK-3β/GSK-3β ratio (Figures 2E, F) and increase cleaved-caspase-1 expression (Figures 2E, G) at the protein level. Importantly, we found that ticagrelor increased the p-GSK-3β/GSK-3β ratio and cleaved-caspase-1 by Dox.

### Ticagrelor reduces the expression of IL-1β and IL-18 in Dox treated rats

**FIGURE 3:**
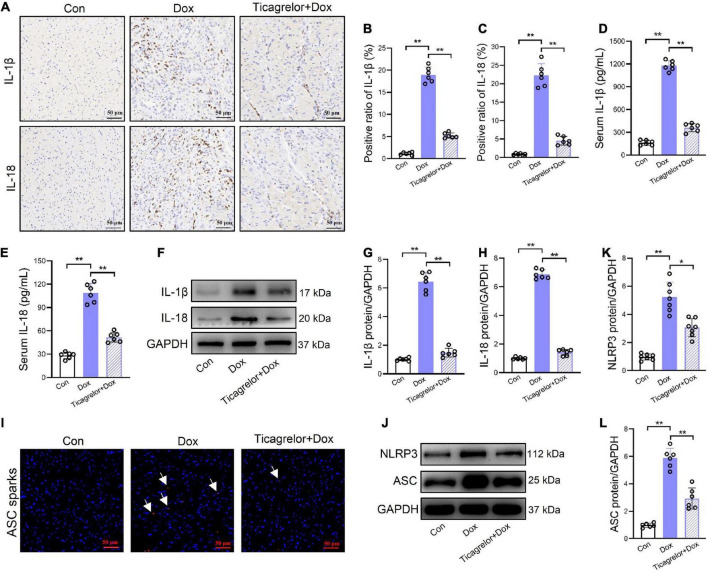
Ticagrelor reduces the expression of IL-1β and IL-18 in Dox treated rats. **(A–C)** Representative images and positive ratio of IL-1β **(A,B)** and IL-18 **(A,C)** under immunohistochemistry (*n* = 6). **(D,E)** IL-1β and IL-18 content in serum of rats (*n* = 6). **(F–H)** Representative immunoblots and the corresponding quantification of IL-1β and IL-18 in heart tissues (*n* = 6). **(I)** ASC sparks in rats heart slices. **(J–L)** Representative immunoblots and the corresponding quantification of NLRP3 and ASC in H9c2 cells assessed using western blot (*n* = 6). *p < 0.05, **p < 0.01.

After GSDMD-NT forms plasma membrane pores, cells secrete IL-1β and IL-18 (15). In rat heart slices, Dox increased the expression of IL-1β and IL-18, and the addition of ticagrelor inhibited the induction of Dox (Figure 3A). Quantitative results also showed that ticagrelor reduced the Dox-induced high expression of IL-1β from 18.9 to 5.2% (Figure 3B) and IL-18 from 22.3 to 4.5% (Figure 3C). ELISA was used to determine the levels of IL-1β and IL-18 in rat serum, which showed that ticagrelor reduced the excessive secretion of IL-1β (Figure 3D) and IL-18 (Figure 3E) induced by Dox. Similarly, the expression of IL-1β (Figures 3F, G) and IL-18 (Figures 3F, H) proteins in the rat heart tissue was promoted by Dox and decreased by ticagrelor. The release of IL-1β and IL-18 is closely related to the activity of NLRP3 inflammasome. We detected the ASC sparks in the rats heart tissue slices, which indicates the activation of NLRP3 inflammasome. The results showed that Dox increase the number of ASC sparks and ticagrelor inhibit the formation of ASC sparks (Figure 3I). Similarly, Dox increased the protein expression of NLRP3 inflammasome components in rats heart, while ticagrelor preconditioning decreased their abnormal increase (Figures 3J–L).

### Ticagrelor inhibits GSK-3β-mediated pyroptosis in RCMs and H9c2 cells treated by Dox

**FIGURE 4:**
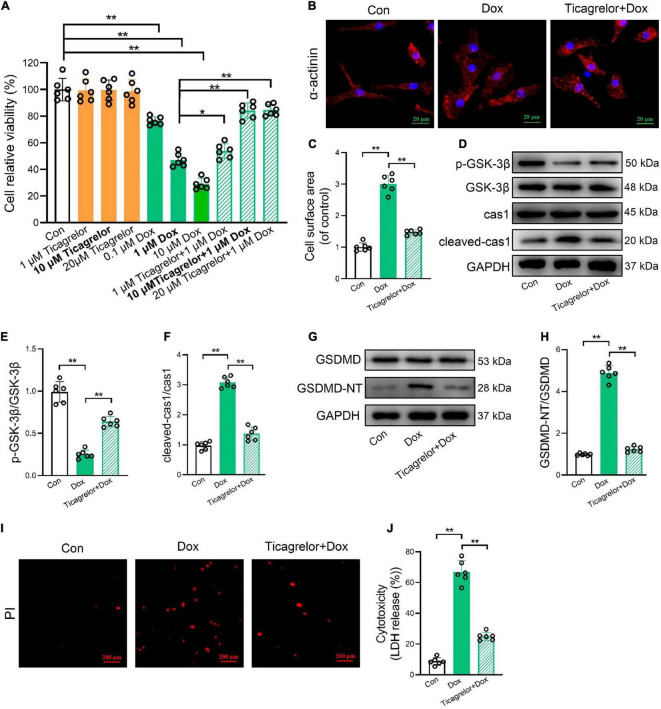
Ticagrelor inhibits GSK-3β-mediated pyroptosis in RCMs treated by Dox. **(A)** Impact of Dox and ticagrelor on RCMs viability (*n* = 6). **(B,C)** Immunofluorescence staining was performed for α-actinin in RCMs and the cell surface area was calculated (*n* = 6). **(D–F)** Representative immunoblots and the corresponding quantification of the ratio of p-GSK-3β/GSK-3β and cleaved-cas1/cas1 in RCMs (*n* = 6). **(G,H)** Representative immunoblots and the corresponding quantification of GSDMD-NT/GSDMD in RCMs. **(I,J)** Representative images of RCMs stained with PI and percentages of pyroptotic RCMs determined using the LDH release assay (*n* = 6). *p < 0.05, **p < 0.01.

We verified the effect of ticagrelor on Dox-induced pyroptosis in RCMs and H9c2 cells. Viability was evaluated using the MTT assay. Under normal conditions, ticagrelor at a concentration of 20 μm did not reduce cell viability over 24 h. A dose-dependent increase in cell viability was observed following 1 h pretreatment with ticagrelor (1, 10, and 20 μm) and Dox (0.1, 1, and 10 μm) (Figure 4A). A concentration of 10 μm ticagrelor and a concentration of Dox (1 μm) were used for the subsequent *in vitro* experiments. The immunofluorescence results for α-actinin showed that Dox significantly increased the surface area of RCMs and could be inhibited by ticagrelor (Figures 4B, C). Western blot analysis showed that the p-GSK-3β/GSK-3β ratio was decreased (Figures 4D, E), whereas cleaved-caspase-1 expression (Figures 4D, F) and GSDMD-NT were increased (Figures 4G, H) in the Dox group. Ticagrelor inhibited changes in these proteins. Moreover, ticagrelor reduced the number of PI-positive (Figure 4I) and LDH-releasing cells (Figure 4J) upon Dox stimulation.

**FIGURE 5:**
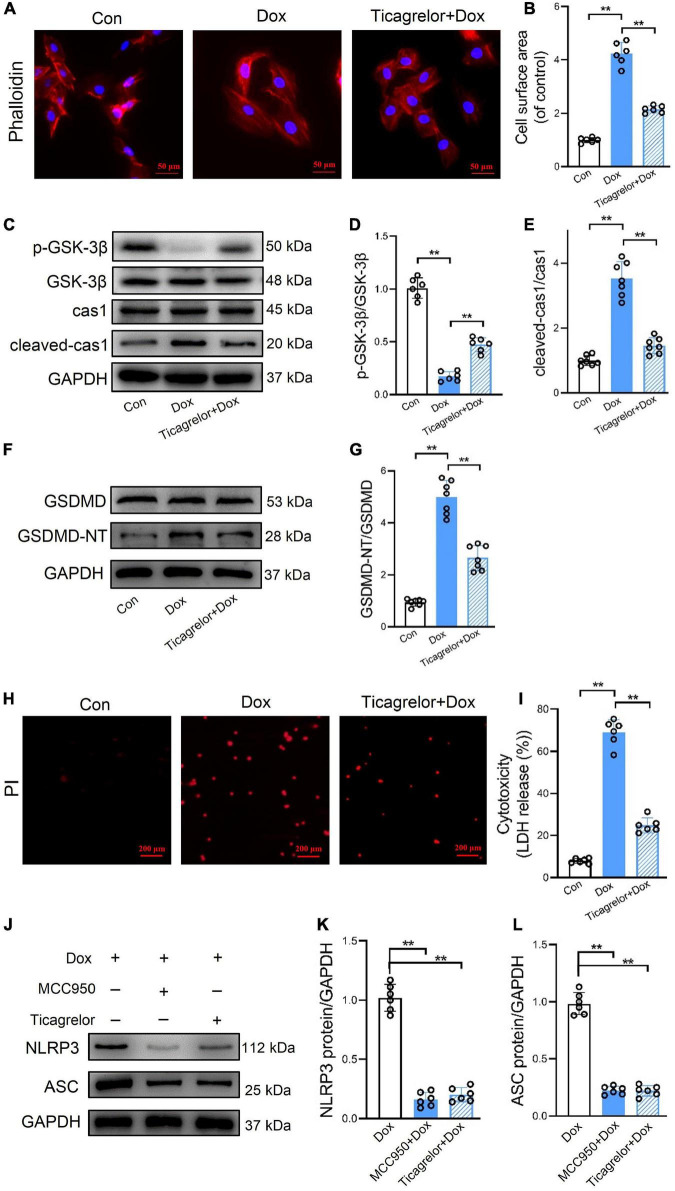
Ticagrelor inhibits GSK-3β-mediated pyroptosis in H9c2 cells treated by Dox. **(A,B)** Representative images of H9c2 cells stained with phalloidin and the cell surface area was calculated (*n* = 6). **(C–E)** Representative immunoblots and the corresponding quantification of the ratio of p-GSK-3β/GSK-3β and cleaved-cas1/cas1 in H9c2 cells (*n* = 6). **(F,G)** Representative immunoblots and the quantification of GSDMD-NT/GSDMD in H9c2 cells (n = 6). **(H,I)** Representative images of H9c2 cells stained with PI and percentages of pyroptotic H9c2 cells (*n* = 6). **(J–L)** Representative immunoblots and the quantification of NLRP3 and ASC in H9c2 cells assessed using western blot (*n* = 6). **p < 0.01.

Similar to the above results in RCMs, ticagrelor countered the cell surface area of H9c2 cells (Figures 5A, B), the p-GSK-3β/GSK-3β ratio (Figures 5C, D), and the expression of cleaved-caspase-1 expression (Figures 5C, E) and GSDMD-NT (Figures 5F, G) proteins induced by Dox in H9c2 cells. Ticagrelor also reduced the number of PI-positive (Figure 5H) and LDH-releasing cells (Figure 5I) after Dox stimulation. Although the effect of Dox on pyroptosis is increasingly recognized, DOX-induced apoptosis is still the main mechanism of cell death. We measured cleaved caspase-3 and cleaved PARP in rats heart, RCMs and H9c2 cells to confirm this (Supplementary Figure 1). Ten micrometers MCC950 showed no effects on cell viability and cytotoxicity (Supplementary Figure 2). We added MCC950 and ticagrelor, respectively, before adding Dox, and finally detected the expression changes of NLRP3 and ASC (Figures 5J–L). The results showed that both MCC950 and ticagrelor prevent the deleterious action of Dox.

### GSK-3β/caspase-1/GSDMD pathway plays a role in Dox-induced pyroptosis in H9c2 cells

**FIGURE 6:**
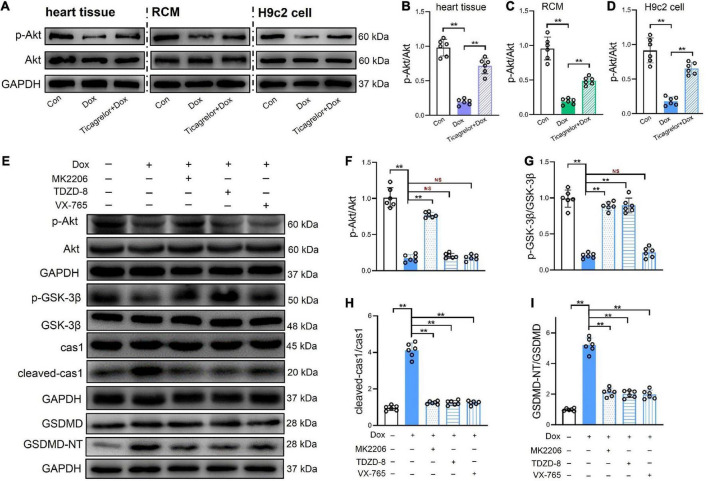
GSK-3β/caspas-1/GSDMD pathway plays a role in Dox induced pyroptosis of H9c2 cells. **(A–D)** Representative immunoblots and the corresponding quantification of the ratio of p-Akt/Akt in heart tissues, RCMs and H9c2 cells (*n* = 6). **(E–I)** Representative immunoblots and the corresponding quantification of the ratio of p-Akt/Akt, the ratio of p-GSK-3β/GSK-3β, cleaved-cas1/cas1, and GSDMD-NT/GSDMD in H9c2 cells (*n* = 6). **p < 0.01.

The *in vivo* and *in vitro* results of this study indicate that Dox decreases the content of phosphorylated GSK-3β at serine 9, which seems to be related to pyroptosis in H9c2 cells. Akt inactivates GSK-3β by phosphorylating it at serine 9 (22). The effect of Dox on Akt expression was also investigated. As shown in Figure 6, the ratio of p-Akt (Ser473)/Akt in rat heart tissue (Figures 6A, B), RCMs (Figures 6A, C), and H9c2 cells (Figures 6A, D) was suppressed by Dox stimulation, and ticagrelor increased the phosphorylated level of Akt. To further verify the role of the GSK-3β/caspase-1/GSDMD pathway in Dox-induced pyroptosis of H9c2 cells, the Akt inhibitor MK2206 (10 μm), GSK-3β inhibitor TDZD-8 (20 μm) and caspase-1 inhibitor VX-765 (25 μm) were applied to H9c2 cells. According to the quantitative analysis of proteins in Figure 6E, the results showed that inhibition of Akt, GSK-3β and caspase-1 effectively alleviated the Dox-induced increase in GSDMD-NT in H9c2 cells, that is, it alleviated pyroptosis in H9c2 cells. Compared with the DOX group, MK2206 affected not only the p-Akt/Akt ratio but also the p-GSK-3β/GSK-3β ratio and cleaved-caspase-1. TDZD-8 altered the p-GSK-3β/GSK-3β ratio and cleaved-caspase-1. However, VX-765 did not interfere with the ratios of p-Akt/Akt and p-GSK-3β/GSK-3β (Figures 6F–I). Therefore, in Dox-induced cardiotoxicity, we speculate that GSK-3β plays a regulatory role upstream of caspase-1 and that the pathway could be GSK-3β/caspase-1/GSDMD.

### GSK-3β knockdown diminishes the palliative effect of ticagrelor on Dox induced pyroptosis

**FIGURE 7:**
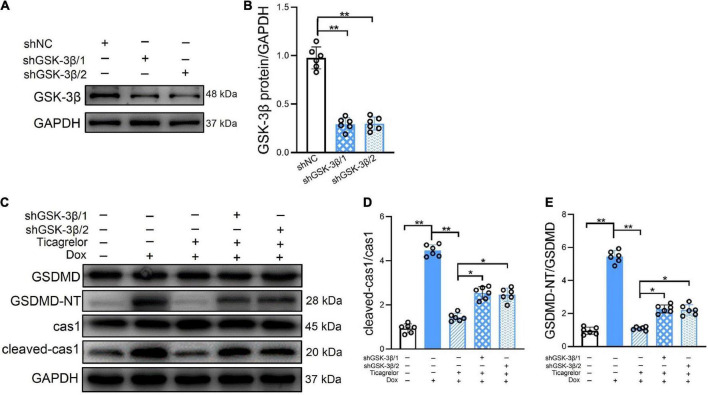
GSK-3β knockdown diminishes the effect of ticagrelor on Dox induced pyroptosis of H9c2 cells. **(A,B)** The shGSK-3βs efficiency in H9c2 cells were examined using western blot (*n* = 6). **(C–E)** The ratios of cleaved-cas1/cas1 and GSDMD-NT/GSDMD in shRNAs-treated H9c2 cells assessed using western blot (*n* = 6). *p < 0.05, **p < 0.01.

If the effect of ticagrelor in alleviating Dox-induced pyroptosis is mediated by GSK-3β, how will knockdown of GSK-3β influence the effect of ticagrelor? We used two significant shRNAs to knock down GSK-3β in H9c2 cells (Figures 7A, B), after which ticagrelor was added for pretreatment and DOX was incubated for 24 h. The expressions of cleaved-caspase-1 and GSDMD-NT were detected using western blotting (Figure 7C). The results showed that shGSK-3β 1/2 weakened the inhibitory effect of ticagrelor on cleaved-caspase-1 (Figure 7D) and GSDMD-NT (Figure 7E).

**FIGURE 8:**
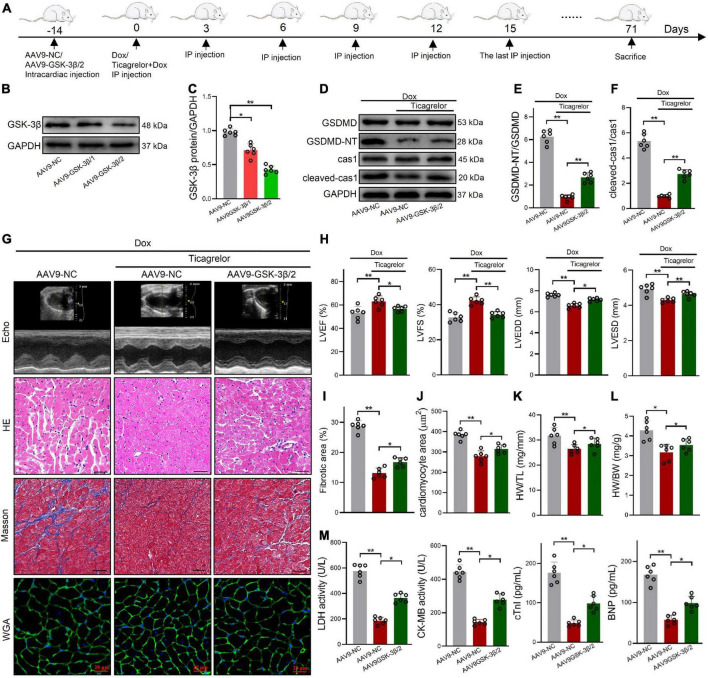
AAV9-GSK-3β diminishes the effect of ticagrelor on Dox induced pyroptosis of rats. **(A)** Schematic illustration of Dox induced cardiac toxicity in AAV9-NC/AAV9-GSK-3β rats treated with ticagrelor pre-administration. **(B,C)** The AAV9-GSK-3β efficiency in rat hearts (*n* = 6). **(D–F)** The ratios of cleaved-cas-1/cas1 and GSDMD-NT/GSDMD in AAV9-GSK-3β-treated rats after Dox administration assessed using western blot (*n* = 6). **(G)** Representative Echo, HE, Masson’s and WGA staining images. **(H)** Cardiac function index for LVEF, LVFS, LVESD, and LVEDD (*n* = 6). **(I)** Fibrosis area of rat heart sections (*n* = 6). **(J)** Quantification of the cardiomyocyte hypertrophy (*n* = 6). **(K,L)** The ratios of HW/BW **(K)** and HW/TL **(L)** in rats (*n* = 6). **(M)** LDH, CK-MB, cTnI, and BNP levels in rats serum (*n* = 6). *p < 0.05, **p < 0.01.

*In vivo*, shGSK-3β/1 and shGSK-3β/2 were injected into rat hearts using the AAV9 vector (Figure 8A). After verification by western blotting, the shGSK-3β/2 sequence was selected (Figures 8B, C). Consistent with the results of the cell experiments, knockdown of GSK-3β in the rat heart reduced the potency of ticagrelor to reduce cleaved-caspase-1 and GSDMD-NT protein (Figures 8D–F). In addition, we evaluated cardiac function (Figures 8G, H), pathological characteristics (Figure 8I), cardiomyocytes area (Figure 8J), the ratios of HW/BW (Figure 8K) and HW/TL (Figure 8L), and serum cardiac-specific parameters (Figure 8M) of the rats in each group. The efficacy of ticagrelor against Dox-induced cardiac injury has been evaluated previously. These results showed that knockdown of GSK-3β reduces the effect of ticagrelor, which is specifically manifested by the reduction of LVEF and LVFS, and the recovery of LVEDD, LVESD, fibrotic area, cardiomyocytes area, LDH, CK-MB, cTnI, BNP, HW/BW, and HW/TL.

## Discussion

Dox is commonly used to treat many tumors such as Kaposi’s sarcoma, acute lymphoblastic leukemia, breast cancer, lymphoma, and bladder cancer (23, 24). Cardiotoxicity cannot be eliminated because Dox does not exhibit cell selectivity. In addition to dexrazoxane, metformin, and eglitaxel have also shown therapeutic effects against Dox-induced cardiotoxicity (25, 26). The search for new strategies that can effectively prevent the cardiotoxic effects of Dox in cancer has been emphasized. In our study, the rats were injected with a cumulative dose of 15 mg/kg Dox or vehicle via three weekly injections (2.5 mg/kg i.p. once every 3 days). Subsequent analyses were performed 8 weeks after the last injection. The main finding of this study indicated that ticagrelor could ameliorate Dox-induced cardiotoxicity by targeting the GSK-3β/caspase-1 pathway.

Defects in cardiomyocyte function are the primary cause of Dox-induced cardiotoxicity. Activation of the NLRP3 inflammasome and pro-inflammatory pyroptosis contribute to Dox-induced dilated cardiomyopathy (27). MCC950 has been proposed as a specific small molecule inhibitor that can impede IL-1β maturation and release through inhibiting the activation of NLRP3 inflammasome (28). Canakinumab is a humanized monoclonal antibody specific for neutralizing IL-1β. The pilot study of CANTOS trial phase II showed that the half-life of canakinumab is very long, and it only needed to be administered once every 12 weeks (29). Furthermore, ticagrelor inhibits the NLRP3 inflammasome to protect against inflammatory diseases independent of the P2Y_12_ signaling pathway (30, 31). In Ye et al. study, ticagrelor (10 or 30 mg/kg) was given via intraperitoneal injection and rats received ticagrelor acute (intraperitoneal; 30 mg/kg), chronic (oral; 300 mg/kg per day) for 4 weeks or the combination (acute + chronic) (32). In our experiment, intraperitoneal injection of ticagrelor into rats at a dose of 7.5 mg/kg has significantly improved the Dox induced cardiotoxicity, which was actually lower than the reported by Ye et al. And we did not increase the dose in order to prevent bleeding events. Our results indicated that Dox increased the protein expression of NLRP3 inflammasome components in rats heart, while ticagrelor preconditioning decreased their abnormal increase. MCC950 and ticagrelor prevent the deleterious action of Dox. At the same time, Dox induces NLRP3 inflammasome activation in rat cardiomyocytes and then leads to pyroptosis. Ticagrelor preconditioning *in vivo* and *in vitro* effectively reduced the cardiotoxicity caused by Dox through inhibiting NLRP3 inflammasome activation.

GSK-3β, a glycogen synthesis kinase, is involved in the occurrence and development of Dox-induced HF. Clinical research showed treatment with the specific inhibitor AR-A14418 aggravates the fibrotic phenotype of systemic sclerosis fibroblasts via inhibition of GSK-3β (33). Another GSK-3 inhibitor, Tideglusib, made it to phase II clinical trial for the treatment of AD and progressive supranuclear palsy (34). GSK-3β deficiency inactivated NLRP3 inflammasome-mediated pyroptosis in LPS-treated periodontal ligament cells (35). The above background research and our experimental results suggested that GSK-3β may be related to Dox induced NLRP3 inflammasome-mediated pyroptosis. Then, we found that GSK-3β regulates cardiomyocyte pyroptosis upstream of caspase-1 and plays an indispensable role in Dox-induced cardiac injury. The beneficial effect of ticagrelor in alleviating Dox toxicity was mediated by the inhibition of GSK-3β *in vivo* and *in vitro*. Therefore, when the protein expression of GSK-3β in rat heart specifically knocked down, the inhibitory effect of ticagrelor preconditioning on Dox-induced cardiotoxicity was weakened.

Overall, our work demonstrates a new pharmacological function of ticagrelor: inhibition of pyroptosis in cardiomyocytes through GSK-3β. These findings may provide a new approach for preventing Dox-induced cardiotoxicity.

## Conclusion

**FIGURE 9:**
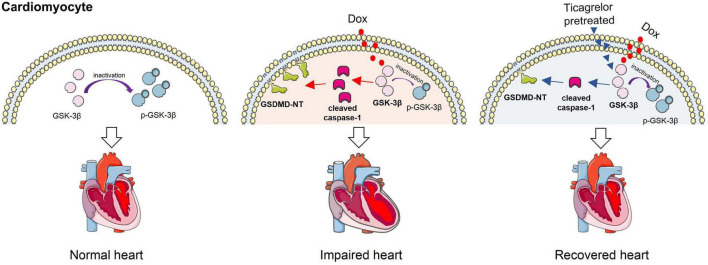
Ticagrelor reduces doxorubicin-induced pyroptosis of rat cardiomyocytes *via* targeting GSK-3β/caspase-1.

This study found that ticagrelor reduced Dox-induced pyroptosis in rat cardiomyocytes by targeting the GSK-3β/caspase-1 pathway, thus alleviating cardiac dysfunction (Figure 9). Therefore, ticagrelor may be a promising therapeutic agent for the cardiotoxic reaction of Dox.

## Data availability statement

The raw data supporting the conclusions of this article will be made available by the authors, without undue reservation.

## Ethics statement

The animal study was reviewed and approved by the Animal Experiments Committee of Zhengzhou University.

## Author contributions

S-HW, S-NH, and QL conceived and designed the experiments. S-HW, M-JS, S-YD, C-LL, J-MW, and XL performed the experiments. S-HW, M-JS, and S-YD analyzed the data. S-HW, XL, and QL contributed to the writing of the manuscript. All authors contributed to the article and approved the submitted version.

## Supplementary material

Supplementary Figure 1 Dox induced cardiomyocytes apoptosis. **(A–D)** Representative immunoblots and the corresponding quantification of the ratio of cleaved-PARP/PARP and cleaved-cas3 in heart tissues, RCMs and H9c2 cells (*n* = 6).

Supplementary Figure 2 MCC950 hardly damages RCMs or H9c2 cells. Impact of Dox and ticagrelor on RCMs and H9c2 cells viability (*n* = 6).
