# Supplementary material for: Ticagrelor reduces doxorubicin-induced pyroptosis of rat cardiomyocytes by targeting GSK-3β/caspase-1
Source: Front Cardiovasc Med. 2023 Jan 6;9:1090601. doi: 10.3389/fcvm.2022.1090601 (PMC9853199; doi:10.3389/fcvm.2022.1090601)
Supplement: Supplementary file 1 [file Table_1.DOCX]

|  | At the start point | | At the end point | |
| --- | --- | --- | --- | --- |
|  | HW (mg) | BW (g) | HW (mg) | BW (g) |
| Con | - | 221.1±5.4 | 850.0±136.0 | 268.4±11.1 |
| Dox | - | 219.3±6.4 | 1111.7±104.0^*^ | 260.1±11.4 |
| Ticagrelor/ Dox | - | 220.8±4.0 | 881.7±83.7^#^ | 267.3±9.4 |
| AAV9-NC/Dox | - | 220.0±6.0 | 1118.3±117.4 | 261.4±11.8 |
| AAV9-NC/ Ticagrelor/Dox | - | 222.5±6.6 | 825.0±117.4^&^ | 261.0±4.5 |
| AAV9-GSK-3β/2/ Ticagrelor/Dox | - | 222.3±5.7 | 876.7±88.4^$^ | 265.6±7.1 |

**Table S1. Heart weight and body weight of the experimental animals (mean±SD, n = 6)**

^*^*p* < 0.05 *vs* Con, ^#^*p* < 0.05 *vs* Con, ^&^*p* < 0.05 *vs* AAV9-NC/Dox, ^&^*p* < 0.05 *vs* AAV9-NC/ Ticagrelor/Dox.
