# Supplementary figures and images for: Ticagrelor reduces doxorubicin-induced pyroptosis of rat cardiomyocytes by targeting GSK-3β/caspase-1
Source: Front Cardiovasc Med. 2023 Jan 6;9:1090601. doi: 10.3389/fcvm.2022.1090601 (PMC9853199; doi:10.3389/fcvm.2022.1090601)

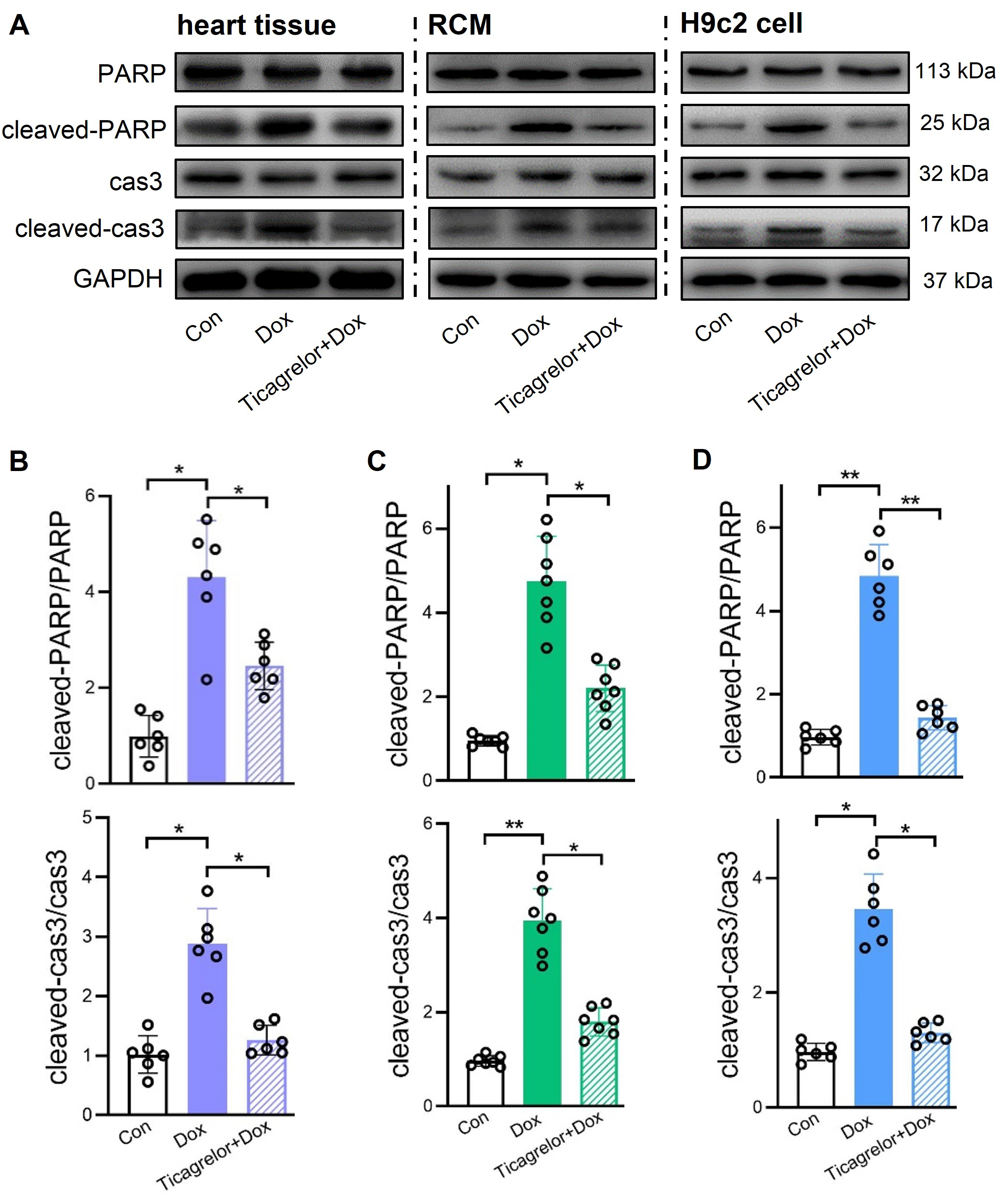

Supplement: Supplementary Figure 1 — Dox induced cardiomyocytes apoptosis. (A–D) Representative immunoblots and the corresponding quantification of the ratio of cleaved-PARP/PARP and cleaved-cas3 in heart tissues, RCMs and H9c2 cells (n = 6). [file Image_1.jpeg]

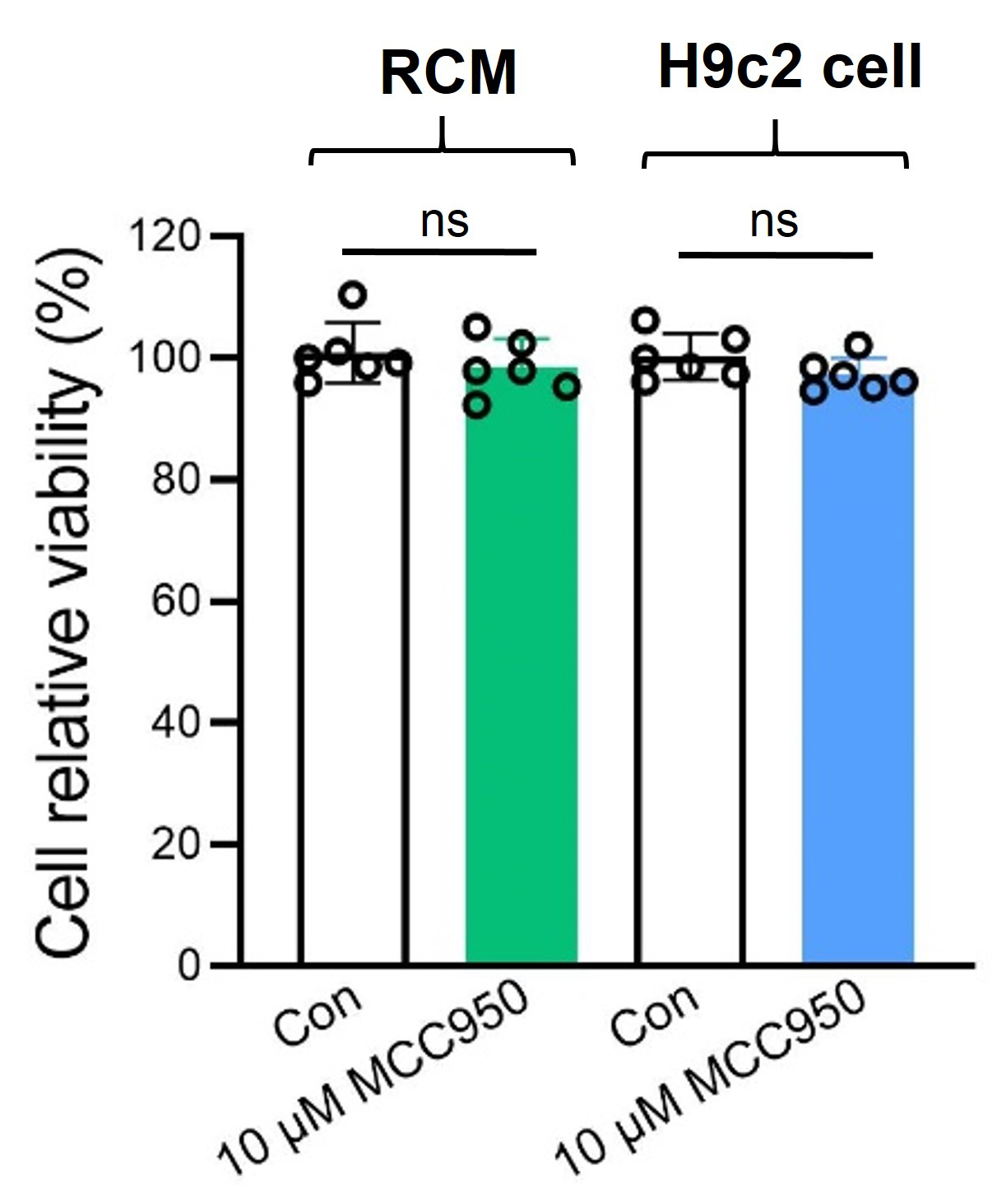

Supplement: Supplementary Figure 2 — MCC950 hardly damages RCMs or H9c2 cells. Impact of Dox and ticagrelor on RCMs and H9c2 cells viability (n = 6). [file Image_2.jpeg]
